# What is the role of occupational physicians in the workplace during the COVID-19 pandemic in Japan? A qualitative interview study

**DOI:** 10.1186/s12913-022-08659-y

**Published:** 2022-10-27

**Authors:** Yu Igarashi, Seiichiro Tateishi, Tomoko Sawajima, Kodai Kikuchi, Mika Kawasumi, Juri Matsuoka, Arisa Harada, Koji Mori

**Affiliations:** 1 Disaster Occupational Health Center, Institute of Industrial Ecological Sciences, University of Occupational and Environmental Health, 1-1 Iseigaoka, Yahatanishi-ku, 807-8555 Kitakyushu, Japan; 2 Shizuoka Health Care Office, Health Care Center, Central Japan Railway Company, Shizuoka, Japan; 3 Department of Occupational Health Practice and Management, Institute of Industrial Ecological Sciences, University of Occupational and Environmental Health, Kitakyushu, Japan; 4 Ricoh Company, Ltd, Tokyo, Japan; 5 Hoya Corporation, Tokyo, Japan; 6 Department of Occupational Medicine, School of Medicine, University of Occupational and Environmental Health, Kitakyushu, Japan

**Keywords:** COVID-19, Disaster, Pandemic, Emerging infectious diseases, Occupational health and safety, Occupational physician, Qualitative interview study

## Abstract

**Background:**

The coronavirus disease 2019 (COVID-19) pandemic has had various impacts on businesses and workers worldwide. The spread of infection has been reported through cluster outbreaks in the workplace, and World Health Organization has emphasized workplace infection control measures. Occupational physicians (OPs) are expected to actively support employers’ efforts to minimize the damage of the pandemic. However, there is little research on the role of these specialists during a pandemic. Clarification of the contributions of OPs to health and safety at the workplace in the COVID-19 pandemic would be beneficial to ensure that OPs can be effectively deployed in the next pandemic.

**Methods:**

We employed semi-structured interviews and qualitative content analysis of the interview transcripts. Twenty OPs were selected as priority candidates from among 600 OPs certificated of the JSOH, and thirteen who met the eligibility criteria agreed to participate. The online interviews were conducted in November and December 2020 with thirteen OPs. We extracted meaning units (MUs) from interview transcripts according to the research question: “What was the role of OP in the COVID-19 pandemic?“ and condensed and abstracted them into codes and categorized them. Validity was confirmed by additional 5 OPs interviews.

**Results:**

A total of 503 MUs were extracted from the transcripts. These were abstracted into 10 sub-categories and two categories. Categories 1 and 2 dealt with “Role in confronting the direct effects of the pandemic” and “Role in confronting the indirect effects of the pandemic” and accounted for 434 (86.3%) and 69 (13.7%) MUs, respectively. These results were validated by another 5 interviews.

**Conclusion:**

This study identified the role of OPs in Japan in the COVID-19 pandemic. The results showed that they made a wide range of contributions to the direct and indirect effects of the pandemic. We hope our findings will help OPs during future pandemics or other long-term emergency situations.

## Background

Workplaces, where large numbers of people work, are the primary sites for spreading the coronavirus disease 2019 (COVID-19) pandemic [1, 2], and World Health Organization, International Labour Organization, Centers for Disease Control and Prevention, and other organizations have indicated the need for countermeasures to address the situation [3–6]. COVID-19 control measures in the workplace include wearing masks, ventilation, hand washing, telecommuting, and online conferencing [3–6]. The involvement of occupational health and safety (OHS) professionals such as occupational physicians (OPs) is necessary to implement appropriate COVID-19 countermeasures [6].

The COVID-19 pandemic has caused various health problems for workers in companies and other workplaces besides the infection itself [7–9]. These are secondary health problems such as musculoskeletal disorders due to teleworking at home in an inadequate environment and mental health problems due to isolation, lack of support, and overwork [10–14]. Accumulated stress and fatigue from teleworking can reduce work accuracy, increase the potential for human error, and increase the risk of workplace accidents and incidents, which can potentially be a problem for business continuity [15]. In the risk tradeoff between psychological burden and infectious disease, the involvement of OPs may be the core of the process for the decision-making process of the business [16].

OPs are expected to play a role in supporting businesses in taking appropriate measures to fulfill their responsibility to secure workers’ health and preserve the stability of business operations, and this is also the case in the COVID-19 pandemic. There have been several reports worldwide regarding OHS functions during the COVID-19 pandemic, including expert opinions on the role of the OP and their actual response to the workers’ health problems [14, 17–23]; a literature review of OPs’ role [24]; a questionnaire survey of OPs in the United Kingdom on how OHS services have changed in practice [25]; a questionnaire survey of OPs in Japan on useful information in the COVID-19 pandemic [26]; a report on OHS activities in a Singapore hospital [27] and reports on the role of Italian OPs in vaccination [28, 29]. In Japan, the Occupational Health and Safety Law requires employers to appoint OPs in workplaces where ≥ 50 workers are regularly employed [30]. As core professionals in the OHS function, OPs in Japan have been actively supporting employers’ efforts against this pandemic.

Preparedness to quickly and accurately assist in maintaining and promoting workers’ health in the event of a pandemic after COVID-19 will be necessary. Emerging infectious diseases such as SARS (severe acute respiratory syndrome), MERS (Middle East respiratory syndrome), and H1N1 (novel influenza) have emerged, pointing to the possibility of more frequent pandemics in the future [31, 32]. To date, few reports have assessed in detail the actual functioning of OPs during pandemics including COVID-19, most of the available reports are expert opinions or partial activity records [11, 14, 17–29]. Assessing OP’s contribution to health and safety at the workplace during the COVID-19 pandemic would be beneficial to ensure that OHS services can be effectively deployed in the next pandemic. Therefore, we conducted a qualitative study to identify what is the role of OPs in the workplace during the ongoing COVID-19 pandemic.

## Methods

### Study design

The study was exploratory in design, using a qualitative interview and inductive approach. Semi-structured interviews were conducted to clarify the research questions using qualitative content analysis [33].

### Participants

The criteria for the subjects were (1) be certified by the Japan Society for Occupational Health (JSOH), (2) have been actively involved in the company’s infectious disease control measures, and (3) have been involved in decision-making. Variation was ensured by including OPs from different regions and industries. We employed snowball sampling to collect the participants. Twenty OPs were selected as priority candidates from among 600 OPs certified by the JSOH through discussion by the researchers and requested their participation in the study by e-mail. Thirteen who met the eligibility criteria agreed to participate, two refused, five did not respond to the request, and we sent no reminder. Regarding the sample size, we aimed to obtain about 12 participants to ensure validity, referring to the study by Guest, Bunce & Johnson [34], and 13 participants were accepted. Since the interviews were analyzed sequentially and no additional information was deemed available, the primary interview was closed. The results of the primary interviews were analyzed and categorized. To confirm the validity of the primary interview, the same criteria were used to select the subjects, and the validation interviews were conducted with five OPs.

### Data collection

**Fig. 1:**
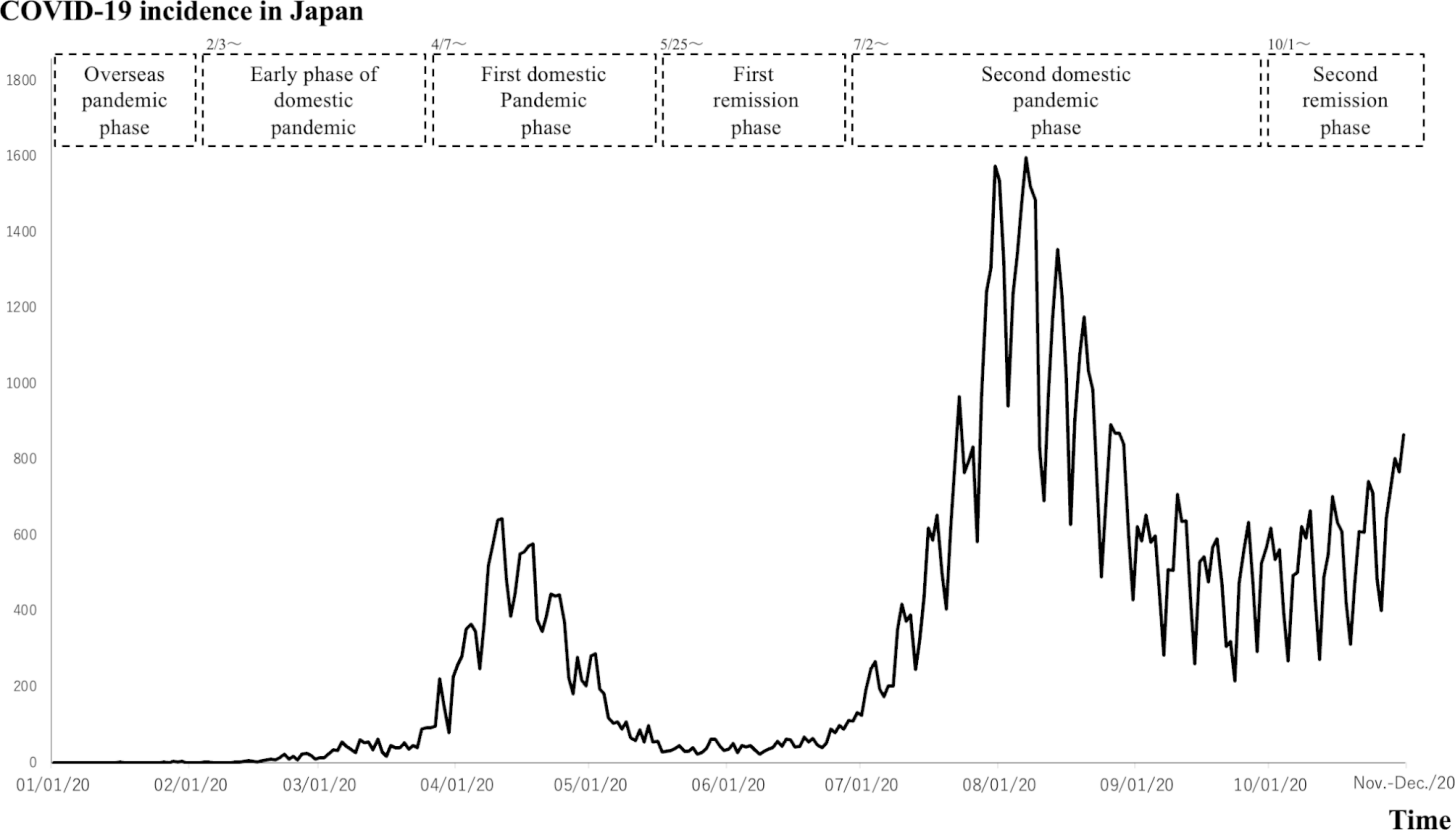
The number of positive COVID-19 incidence in Japan and the Phases in this study. Second remission phase: from Oct. 1, 2020, to the date of interviews conducted in November-December 2020

The online interviews were conducted in November and December 2020 using Zoom videoconferencing software (Zoom Video Communications, Inc. San Francisco, USA). Semi-structured interviews were conducted using an interview guide by two research team members. The two questions of our interview guide protocol were: (1) What were you asked to do by the company? (2) What advice did you give to the company? The “company” was defined as the employer and the personnel in charge of the human resources, safety, and general administration departments and those assigned to be in charge of infection control. We asked participants to answer according to the infection situation in Japan from the time of the outbreak of COVID-19 in China to the time of the interview (Fig. 1). The interviews took between 60 and 90 min to complete, and the audio was recorded. All records of interviews were transcribed for analysis. As compensation for their time, participants each received 10,000 yen. No transcripts were returned to the participants.

Interviews for validation were conducted in August and September 2022 online using Zoom software. We asked participants two protocol questions to answer according to the infection situation in the same time series as a primary interview. Finally, we showed the category table we had created from the primary interview and asked if they had any responses that deviated from the table.

### Data analysis

All members of the research team were OPs. Two (KM, ST) had extensive experience in research and disaster occupational health and supervised the study as senior researchers. Six members (KM, ST, YI, MK, JM, and TS) were board-certified OPs. One author (KK) had four years of experience as an OP. All team members except the least experienced had attended a training course on qualitative research before the study. The expertise and experience of both the researchers and the interviewees helped to ensure the reliability of the data collection and analysis.

The qualitative content analysis was based on the concepts of Graneheim and Lundman [33]. According to Graneheim and Lundman [33], the qualitative content analysis is based on the unit of analysis. The most appropriate unit of analysis is the entire interview or observation protocol, which is large enough to be considered as a whole and small enough to be kept in mind as the context for the meaning unit (MU) during the analysis process. In this study, each interview was considered a unit of analysis. The interviews were read through several times to obtain a sense of the whole. From the units of analysis, texts related to the role of OPs in the COVID-19 pandemic were extracted as MUs. Each MU consisted of words, sentences, or paragraphs containing aspects related to each other through content and context. The MU were then condensed and abstracted to codes, while still preserving the core. Next, the differences and similarities in the semantic content of all codes were compared, and codes with commonalities were collected to create subcategories. The same procedure was then followed with increasing levels of abstraction and finally integrated into categories. The analysis process was systematic, but it involved moving back and forth between the whole and parts of the text. Tentative categories due to discrepancies were discussed and corrected by two senior team members (KM, ST). After reflection and discussion, a consensus was reached on how to categorize the codes.

Handling the infection situation in Japan was based on the “Action Plan for Pandemic Influenza” formulated by the Cabinet Secretariat [35] as a timeline focusing on the pandemic. This started with the overseas epidemic period from Dec 2019 through March 1, 2020 the early domestic epidemic period from March 2, 2020 through July 3, 2020, and the first domestic epidemic period from July 4, 2020 through May 24, 2020 the 1st remission period from May 25, 2020 through June 1, 2020 the 2nd domestic epidemic period from June 2, 2020 through September 31, 2020 and the 2nd remission period starting October 1, 2020 to the date of interviews conducted in November-December 2020. (Table 1; Fig. 1) [36]. MUs spanning multiple phases were counted by phase.

**Table 1:** Phase classifications for the COVID-19 pandemic in this study

Phase	Infection situation
Overseas pandemic phase Dec. 2019-Feb. 2, 2020	The infection is spreading abroad from Wuhan, China.
Early phase of domestic pandemic Feb. 3, 2020-Apr. 6, 2020	This phase started when the Diamond Princess cruise ship arrived at Yokohama. Infections have occurred in some Japanese. It is possible to trace the history of contacts of all patients through epidemiological surveys.
First domestic pandemic phase Apr. 7, 2020-May. 7, 2020	A state of emergency is declared by the Japanese government. Infections have occurred in several prefectures; it is impossible to trace the history of contacts of patients through epidemiological surveys.
First remission phase May. 25, 2020-Jul. 1, 2020	The state of emergency is lifted by the government. The number of infected people declines and remains at low levels.
Second domestic pandemic phase Jul. 2, 2020-Sep. 31, 2020	Infections are spreading again, and the number of infected people is rising steeply, with the daily number of newly infected people in Tokyo > 100.
Second remission phase Oct. 1, 2020-Nov.-Dec. 2021	The number of newly infected people per day reaches a plateau.

COVID-19: Coronavirus disease 2019

### Ethical considerations

Consent was obtained from the participants. It was understood that consent could be withdrawn at any time, and data were anonymized because they included confidential corporate information such as the incidence of infectious diseases in a particular workplace. The study was conducted with approval from the Research Ethics Review Committee of the University of Occupational and Environmental Health, Japan (Approval No. R2-020).

## Results

Table 2 shows the characteristics of the 13 interviewees and the number of MUs extracted from their interview transcripts. The mean duration of an interview was 73.4 min. The mean experience of OPs was 14.6 years. The participants worked in nine regions of Japan and five industries.

**Table 2:** Characteristics of the interviewees and the number of meaning units (MUs) from their interviews

ID No.	Years of experience as OP	Number of employees	Region	Type of industry	Number of MUs from the interview
I1	12	6,000	A	Manufacturer	66
I2	21	500	B	Education	21
I3	19	500	C	Manufacturer	16
I4	15	4,000	D	Manufacturer	58
I5	10	3,000	A	Manufacturer	19
I6	20	7,000	E	Manufacturer	32
I7	14	5,000	F	Manufacturer	39
I8	12	1,000	G	Manufacturer	49
I9	7	1,000	H	Medicine	29
I10	8	2,500	A	Construction	46
I11	9	3,000	C	Manufacturer	36
I12	22	2,000	E	Manufacturer	40
I13	21	25,000	I	Retail	52
				Total	503

MU: Meaning unit; OP: Occupational physician; Region: Prefecture in which the company is located

A total of 503 MUs were extracted from the interview transcripts, condensed and abstracted to codes, and sorted into 10 sub-categories. These sub-categories were classified into two categories: “Role in confronting the direct effects of the pandemic” and “Role in confronting the indirect effects of the pandemic.“ Table 3 describes a theme, categories, sub-categories, and a sample of codes. Finally, there were 434 codes (86.3%) for direct and 69 (13.7%) for indirect effects. Table 4 shows the number of MUs for each sub-category, category, and phase. In the interviews for validation, no responses that deviated from the category we created from primary interviews were heard from the five subjects.

**Table 3:** Theme, categories, sub-categories, and a sample of codes

Theme	Category	Sub-category	A sample of codes
The role of the OP in the COVID-19 pandemic			
	Role in confronting the direct effects of the pandemic		
		Collecting and providing information on the nature of the pathogens and the infection situation	“I sent daily reports to the task force on the number of cases and the status of the epidemic in the region.“(I4) “I provided the task force with information about government and academic guidelines and guided them to act based on the correct information.“ (I1)
		Establishment and participating in the task force and document preparation and revision	“I recommended that an infection control task force be set up as soon as possible.“(I2) “At the direction of the human resources director, I supervised the content of the documents sent to employees.“(I4)
		Advice on improving the work environment to reduce the risk of infection	“I suggested installing plastic curtains at the cash registers.“(I6) “I was asked about ventilation, the material, and height of the partitions between the desks in offices.“(I2)
		Rulemaking and case consultation services to prevent the introduction of infected individuals into the workplace	“I made internal rules about the criteria for staying at home for those who had had close contact.“(I7) “I advised on the content of the internal notice that says, ‘If you have any of these symptoms, please go to the hospital.’“(I3)
		Advice on modifying tasks according to infection risk	“I suggested promoting telecommuting.“(I2) “I advised allowing employees to commute to work in their vehicles.“(I4)
		Providing information to employees on individual infection control measures	“I explained to the employees that I encourage them to wash their hands because disinfectant was unavailable.“(I10) “I was asked to produce a video, so I made a video about proper individual infection control measures.“(I4)
	Role in confronting the indirect effects of the pandemic		
		Psychosocial factors	“I lobbied internally for an anti-discrimination policy.“(I13) “I made and disseminated self-care materials of mental health and stress care.“(I11)
		Ergonomic factors	“I distributed exercise videos for those who had to stay home.“(I2) “I explained work postures and work environments, including overall lighting levels and hand heights for employees working at home.“(I4)
		Physical factors	“A client asked me for advice on how to prevent heatstroke when wearing masks.“(I11)
		Individualized health support	“I wrote prescriptions for employees in countries where customs had stopped.“(I13) “I explained the risks to employees with infectious disease concerns and had them see a doctor.“(I6)

COVID-19: Coronavirus disease 2019, OP: Occupational physician

**Table 4:** Number of meaning units (MUs) of each phase

Category	Sub-category	Overseas pandemic phase	Early phase of domestic pandemic	First domestic pandemic phase	First remission phase	Second domestic pandemic phase	Second remission phase	Total number of MUs Dec. 2019 to Nov.-Dec. 2020
Role in confronting the direct effects of the pandemic								434
	Collecting and providing information on the nature of the pathogens and the infection situation	10	16	13	11	12	10	72
	Establishment and participation in the task force and preparation and revision of documents	12	25	17	11	12	10	87
	Advice on improving the work environment to reduce the risk of infection	2	11	9	7	10	3	42
	Rulemaking and case consultation services to prevent the introduction of infected individuals into the workplace	9	35	24	15	20	16	119
	Advising on modifying of tasks according to infection risk	3	24	22	11	6	8	74
	Providing information to employees on individual infection control measures	7	16	7	4	2	4	40
Role in confronting the indirect effects of the pandemic								69
	Psychosocial factors	0	5	18	7	11	5	46
	Ergonomic factors	0	4	6	1	1	1	13
	Physical factors	0	1	0	2	1	0	4
	Individualized health support	3	1	1	0	0	1	6
Total		46	138	117	69	75	58	503

MU: Meaning unit

### Role in confronting the direct effects of the pandemic

According to the interviewees’ experience, their actions against the direct effects of the COVID-19 pandemic included the following six “sub-categories”:

1. Collecting and providing information on the nature of the pathogens and the infection situation.
2. Establishment and participation in the task force and preparation and revision of documents.
3. Advice on improving the work environment to reduce the risk of infection.
4. Rulemaking and case consultation services to prevent the introduction of infected individuals into the workplace.
5. Advice on modifying tasks according to infection risk.
6. Providing information to employees on individual infection control measures.

The actions of the OP were divided into information matters, involvement in company rules and meetings, and advice on the work environment and operations.

### Collecting and providing information on the nature of the pathogens and the infection situation

This sub-category included collecting and providing information for appropriate infection control measures. Information about infectious disease risks and countermeasures were collected from public institutions (i.e., World Health Organization, Centers for Disease Control and Prevention, Ministry of Health, Labour and Welfare), specialized organizations (i.e., Infectious Disease Surveillance Center, JSOH, The Japanese Association for Infectious Diseases), and OPs at other companies. Information was provided to employers and employees. Since the information was updated constantly during the pandemic, it was mentioned in the interviews, not only in the early phases of the pandemic but also in the later phases.

Examples of these codes are as follows:

“I sent daily reports to the task force on the number of cases and the status of the epidemic in the region.“(I4)

”I provided the task force with information about government and academic guidelines and guided them to act based on the correct information.“(I1)

### Establishment and participation in the task force, and preparation and revision of documents

This sub-category included the establishment of an infection control system within the company, such as task forces and regular meetings; participation in management and personnel meetings to discuss infection control measures; preparation and revision of documents, such as the making of a policy and manual for COVID-19 measures; and supervision of public relations documents for external use. In the early phase of the pandemic, a task force was established to formulate rules, which were constantly revised based on the latest guidelines from public agencies and evidence from scientists. In the latter phase, the infection control system was downsized.

Examples of these codes are as follows:

“I recommended that an infection control task force be set up as soon as possible.“(I2)

”At the direction of the human resources director, I supervised the content of the documents sent to employees.“(I4)

### Advice on improving the work environment to reduce the risk of infection

This sub-category included patrolling the work environment to assess the risk of spreading an infection; advice on ventilation, disinfection of contact areas and partitions; and construction of seating arrangements to ensure adequate physical distance to lower the risk of infection in the workplace.

Examples of these codes are as follows:

“I suggested installing plastic curtains at the cash registers.“(I6)

”I was asked about ventilation, the material, and height of the partitions between desks in the offices.“(I2)

### Rulemaking and case consultation services to prevent the introduction of infection into the workplace

This sub-category included rulemaking and case handlings, such as home isolation of infected or exposed persons, return to work, temperature measurement, and hospital visit criteria to prevent the introduction of infection into the workplace. It was reported that infected or exposed persons suspected of being infected could bring infection to the workplace even if they were asymptomatic [37], and guidelines needed to be in place to control the hazard. COVID-19 has a long incubation period, requiring isolation of those who have had contact with infected persons. Therefore, many participants mentioned measures to keep the people who had had contact with the infected person at home. Although the cooperation of employees is essential for infection control measures, together with implementation on the part of the company, this sub-category includes the code for infection control measures implemented by the company.

Examples of these codes are as follows:

“I made internal rules about the criteria for staying at home for those who had had close contact.“(I7)

”I advised on the content of the internal notice that says, ‘If you have any of these symptoms, please go to the hospital.’“(I3)

### Advice on modifying tasks according to infection risk

This sub-category included the implementation of alternative arrangements such as telecommuting, switching to online conferencing, other changes in commuting methods, restrictions on overseas and domestic travel, and whether or not work-related events such as initiation ceremonies could be held. In addition, because pregnant workers and workers with pre-existing medical conditions are at higher risk of becoming seriously ill when infected [38], changes to tasks such as telecommuting and avoiding commuting during congested hours were individualized according to the individual infection risk.

Examples of these codes are as follows:

“I suggested promoting telecommuting.“(I2)

”I advised allowing employees to commute to work in their vehicles.“(I4)

### Providing information to employees on individual infection control measures

This sub-category included anti-infection actions that individuals can take, such as hand sanitization, wearing masks, cough etiquette, daily temperature measurement, and changing non-work-related public activities such as traveling and drinking. Unlike maintenance of the work environment, rulemaking, and changes in tasks, the company has no control over infection control measures that individuals can take, and information imparted to employees about public activities was therefore considered a separate sub-category. Information was provided through the intranet, internal newsletters, e-learning, and training sessions.

Examples of these codes are as follows:

“I explained to the employees that I encouraged them to wash their hands because disinfectant was unavailable.“(I10)

”I was asked to produce a video, so I made a video about proper individual infection control measures. “(I4)

### Role in confronting the indirect effects of the pandemic

OPs took action for the health-related effects on workers that occurred as a result of infection control measures or changes caused by COVID-19. The actions for such indirect effects of the COVID-19 pandemic included the following four “sub-categories”:

1. Psychosocial factors.
2. Ergonomic factors.
3. Physical factors.
4. Individualized health support.

OPs’ actions for the indirect effects of COVID-19 were mainly related to working from home as a change in work style.

### Psychosocial factors

This sub-category included information dissemination, health surveillance, and interviews with workers to prevent health problems caused by psychosocial factors such as isolation and lack of support, overwork in working from home, fear of infection, and discrimination against infected individuals.

Examples of these codes are as follows:

“I lobbied internally for an anti-discrimination policy“(I13)

”I made and disseminated self-care materials on mental health and stress care.“(I11)

### Ergonomic factors

This sub-category included providing information on preventing health problems caused by ergonomic factors such as back pain, stiff shoulders, swollen feet, and headaches due to working from home.

Examples of these codes are as follows:

“I distributed exercise videos for those who had to stay home.“(I2)

”I explained work postures and work environments, including overall lighting levels and hand height for employees working at home.“(I4)

### Physical factors

This sub-category included providing information on preventing health problems caused by the physical factor of heatstroke caused by masks used to avoid infection.

An example of these codes is as follows:

“A client asked me for advice on how to prevent heatstroke when wearing masks.“(I11)

### Individualized health support

This sub-category included providing information on the worsening of chronic diseases due to treatment interruptions or lifestyle changes during COVID-19, support for receiving medical care, and health guidance.

Examples of these codes are as follows:

“I wrote prescriptions for employees in countries where customs had stopped.“(I13)

”I explained the risks to employees with infectious disease concerns and had them see a doctor.“(I6)

## Discussion

In this study, we identified the actual role played by OPs in the COVID-19 pandemic in Japan through a qualitative study. The role played by OPs was mainly categorized into “Role in confronting the direct effects of the pandemic”, which were biological factors, and “Role in confronting the indirect effects of the pandemic”; each includes six and four sub-categories. This study is the first to describe the detailed response of OPs to COVID-19 in Japan. In the event of an emerging infectious disease pandemic such as COVID-19, OPs may respond in a trial-and-error manner. Therefore, the organization of the role of OPs in this study can contribute to their accurate response during the ongoing COVID-19 pandemic and the next pandemic.

### Role in confronting the direct effects of the pandemic

In this study, OP’s actions in confronting the direct effects of the COVID-19 pandemic were converged into six sub-categories described above. Since infection control in the workplace during a pandemic is risk management against pathogens, which are biological factors, ISO 45,001, the international standard for OHS management systems, is a good reference. ISO/PAS 45005:2020 “Occupational health and safety management—General guidelines for safe work during a COVID-19 pandemic” has been published based on ISO 45,001 [39]. The PDCA cycle of “Do - Check - Act” is a system for risk management [40]. In light of the findings of this study, “Collecting and providing information on the nature of the pathogens and the infection situation” can be considered as the collection of information necessary to evaluate risks and opportunities in planning efforts, while “Establishment and participation in the task force, and preparation and revision of documents” corresponds to the support components necessary for effective implementation of the plan. On the other hand, the four actions—“ Advice on improving the work environment to reduce the risk of infection”, “Rulemaking and case consultation services to prevent the introduction of infected individuals into the workplace”, “Advice on modifying tasks according to infection risk,“ and “Providing information to employees on individual infection control measures”—are related to the specific infection control content, and can be regarded as an “Action plan” developed using the results of the risk and opportunity assessment and the “Elimination of hazardous sources and reduction of OHS risks” in the operational phase of the plan. Therefore, the role of OPs in the event of a COVID-19 pandemic can be organized as providing professional information and advice to employers and employees as part of the risk management conducted by the company.

There is a debate on applying the Swiss Cheese Theory and the hierarchy of controls used in OHS measures to control infection under COVID-19 [41, 42]. The idea of the Swiss Cheese Theory is that each Swiss cheese with different hole sizes and locations can be used as a safety measure and that although no single measure can prevent accidents, a series of measures can prevent them [41]. On the other hand, the hierarchy of controls is the concept of prioritizing OHS risk measures based on an understanding of their effectiveness in reducing risk [42]. Generally, effectiveness decreases in the following order: elimination—substitution—engineering controls—administrative controls—personal protective equipment. Multiple measures based on the Swiss Cheese Theory, which has been introduced in infection control, are considered to be effective against COVID-19 in the workplace [43]. However, with the clarification of the nature of COVID-19, which was thought to have three possible routes of infection—contact, droplet, and aerosol—the outbreak of mutant strains such as the highly infectious Delta and Omicron variants showed that the risk of droplet and aerosol exposure was more significant than the risk from contact exposure. As it has become clear that the effectiveness of countermeasures is high, it has become necessary to take effectiveness into account in determining countermeasures; some argue that the application of the Swiss Cheese Theory does not reflect such differences in the effectiveness of infection control measures and that the concept of the hierarchy of controls should be introduced [44].

There was little mention of prioritization in the role of OPs organized in this study, such as “Advice on improving the work environment to reduce the risk of infection”, “Rulemaking and case consultation services to prevent the introduction of infection into the workplace”, “Advice on modifying tasks according to infection risk”, and “Providing information to employees on individual infection control measures” that contribute to infection control. Therefore, we can regard the introduction of the Swiss Cheese Theory approach as a multiplicity of possible measures. The “Guide to Countermeasures for New Coronavirus Infections in the Workplace” [45] by the Japan Society of Travel Medicine and the JSOH, which many OPs in Japan refer to, recommends parallel measures such as environmental measures, behavioral changes in employees, and consideration of those with risk factors for serious illness. Since the study was conducted in November-December 2020 when the nature of COVID-19 was becoming more evident, and since the content of the study concerning advice given by OPs after the outbreak of COVID-19, it is reasonable to assume that they were providing advice and information regarding multiple measures.

### Role in confronting the indirect effects of the pandemic

As a result of, and in the course of, the measures taken to control infection with COVID-19, a variety of health problems arose for workers in addition to infection by COVID-19. The OPs in this study dealt with a wide range of indirect effects of this pandemic. These were converged into four sub-categories, namely three types of factors involving “Psychosocial factors,“ “Ergonomic factors,“ and “Physical factors,“ as well as “Individualized health support” for individual workers’ health concerns and pre-existing conditions, etc.

After the occurrence of the COVID-19 pandemic, many companies rapidly introduced working from home to reduce the risk of infection [46]. Many of these companies introduced the system without prior preparation, which had the potential to cause a variety of problems for workers. The main problems have been psychosocial factors, such as increased stress due to blurred boundaries between work and home and lack of support from supervisors and co-workers, and ergonomic factors, such as musculoskeletal disorders caused by working with fixtures and environments unsuitable for work [10–14]. The COVID-19 pandemic has also caused discrimination and stigma against infected individuals, their families, and those who do not vaccinate [47, 48]. Interviewers heard that OPs were asked to provide information to the company to prevent these problems.

In addition, concerns and consultation with OPs about the increased risk of heatstroke due to wearing masks, as indicated in workplaces where workers work outdoors in the summer, were addressed. The effect of wearing masks on heat dissipation is small [49], and there are no reports of an increase in the number of heatstroke cases. Rather, this concern turned out to be simply a manifestation of concern about wearing masks. OPs are expected to act on such concerns with appropriate evidence.

In the COVID-19 pandemic, treatment interruptions and worsening of chronic diseases have been reported [50, 51] due to concerns about the risk of infection by going out or visiting medical facilities. Since pandemics also bring changes to society as a whole, which can affect lifestyle-related illnesses and health care for chronic diseases, this suggests that OPs are also required to provide individualized health support for such workers.

Expert opinions have been reported on the role of OHS in the COVID-19 pandemic, assessment of the health effect of telework, the survey of useful information for OPs, and reports on OHS activities [11, 14, 17–29]. These are summarized as follows: actions required of OPs include: protecting workers’ health through leadership, collaboration with other occupations and departments, creating a system of worker isolation and return, providing professional information, promoting the use of appropriate protective equipment, temperature measurement and surveillance of symptoms, personalized fitness for work, minimize the adverse effects of telework, and vaccinations. The actions of the OPs observed in this study were consistent, except for vaccination. Regarding vaccination, it was observed that the OP in Japan played the expected role, as OPs administered the vaccination in the subsequent activities. As Spagnolo et al. noted, the role of OPs in a COVID-19 pandemic can vary greatly depending on the social context [24], and it is unclear whether the findings can be applied to the OPs in other countries. However, our findings are applicable to OHS activities worldwide because the infection control measures needed in the workplace are universal and basically the same [3–6].

### Limitations

One limitation of this study is recall bias because we asked about past actions since the COVID-19 outbreak. However, the timing of the study was within the pandemic period of November-December 2020, and we do not believe that recall bias is significant. Second, the 18 interviewees for this study were selected from our specific network and board-certified OP, which have a selection bias, and the results reflect only a subset of OP activities in Japan. However, we believe this study shows an appropriate response by OPs because it comes from the experiences of OPs at a high level of expertise. Although there are limitations in applying the findings of this study to other general-level occupational physicians in Japan, the findings of this study can be used to fill the gap, as it is necessary to train OPs to play an effective role during a pandemic. Third, because our study subjects are from large companies, there are limitations in applying our findings to small and medium-sized companies with smaller OHS resources. Further research would be needed to reflect the situation in other small or medium-sized enterprises, where OHS activities may be less adequate than in large companies. The total sample size (n = 18) does not fully cover all possible roles of OPs, but since a variety of unanticipated events can occur during a pandemic or other disaster, even a large sample size is unlikely to cover all possibilities. The COVID-19 pandemic is not yet under control, and OPs may be required to take on other responsibilities which were not considered in this study. In particular, with the emergence of variant strains, additional research is warranted to determine how OPs can contribute to the strengthening and changing priorities of countermeasures.

## Conclusion

This study identified the role of OPs in Japan in the COVID-19 pandemic. The results showed that they made a wide range of contributions to the direct effects of the pandemic, i.e., the action on biological factors, as well as to the indirect effects of the pandemic.

## Data Availability

The datasets generated and analyzed during the current study are available from the corresponding author on reasonable request.
